# Evaluation of Angiogenesis in an Acellular Porous Biomaterial Based on Polyhydroxybutyrate and Chitosan Using the Chicken *Ex Ovo* Chorioallantoic Membrane Model

**DOI:** 10.3390/cancers14174194

**Published:** 2022-08-30

**Authors:** Zuzana Demcisakova, Lenka Luptakova, Zuzana Tirpakova, Alena Kvasilova, Lubomir Medvecky, Ward De Spiegelaere, Eva Petrovova

**Affiliations:** 1 Department of Morphological Disciplines, University of Veterinary Medicine and Pharmacy in Kosice, Komenskeho 73, 04181 Kosice, Slovakia; 2 Department of Biology and Physiology, University of Veterinary Medicine and Pharmacy in Kosice, Komenskeho 73, 04181 Kosice, Slovakia; 3 Institute of Anatomy, Charles University, U Nemocnice 3, 12800 Prague, Czech Republic; 4 Institute of Materials Research, The Slovak Academy of Sciences, Watsonova 1935/47, 04001 Kosice, Slovakia; 5 Laboratory of Veterinary Morphology, Faculty of Veterinary Medicine, Ghent University, Salisburylaan 133, 9820 Merelbeke, Belgium

**Keywords:** angiogenesis, biomaterial, bone tissue engineering, chitosan, CAM assay, polyhydroxybutyrate, regeneration

## Abstract

**Simple Summary:**

The chorioallantoic membrane (CAM) is an avian extraembryonic membrane widely used as an experimental assay to study angiogenesis and its inhibition in response to tissues, cells, or soluble factors. In recent years, the CAM has become popular in scientific studies focused on the use of its potential for the study of biocompatibility of materials for regenerative strategies and tissue engineering applications. Great research efforts are being made to develop innovative biomaterials able to treat hard tissue defects, including diseases such as a bone cancer. In this article, we describe an approach to detect the formation of blood vessels inside the porous acellular biopolymer polyhydroxybutyrate/chitosan (PHB/CHIT) scaffold using the CAM assay as an in vivo alternative animal model, including macroscopic, histological, immunohistochemical, and molecular evaluation of the biocompatibility.

**Abstract:**

The chorioallantoic membrane (CAM) is a highly vascularized avian extraembryonic membrane widely used as an in vivo model to study angiogenesis and its inhibition in response to tissues, cells, or soluble factors. In recent years, the use of CAM has become an integral part of the biocompatibility testing process for developing biomaterials intended for regenerative strategies and tissue engineering applications. In this study, we used the chicken *ex ovo* CAM assay to investigate the angiogenic potential of innovative acellular biopolymer polyhydroxybutyrate/chitosan (PHB/CHIT) scaffold, which is intended for the treatment of hard tissue defects, depending on treatment with pro- and anti-angiogenic substances. On embryonic day (ED) 7, the experimental biomaterials were placed on the CAM alone or soaked in vascular endothelial growth factor (VEGF-A), saline solution (PHY), or tyrosine kinase inhibitor (SU5402). After 72 h, the formation of vessels was analyzed in the surrounding area of the scaffold and inside the pores of the implants, using markers of embryonic endothelium (WGA, SNA), myofibroblasts (α-SMA), and macrophages (KUL-01). The morphological and histochemical analysis showed strong angiogenic potential of untreated scaffolds without additional effect of the angiogenic factor, VEGF-A. The lowest angiogenic potential was observed in scaffolds soaked with SU5402. Gene expression of pro-angiogenic growth factors, i.e., VEGF-A, ANG-2, and VE-CAD, was upregulated in untreated scaffolds after 72 h, indicating a pro-angiogenic environment. We concluded that the PHB/CHIT has a strong endogenous angiogenic potential and could be promising biomaterial for the treatment of hard tissue defects.

## 1. Introduction

Bone tissue engineering is a branch of regenerative medicine that uses different combinations of scaffolds, biomaterials, seeded cells, and cytokines to treat bone defects. This approach aims to implant engineered bone tissue scaffolds to the bone defect, which are gradually replaced by new bone tissue, thus providing fast healing while also aiming for a durable recovery of the natural biomechanical properties of the bone [1,2,3]. These regenerative strategies can be used in regenerative medicine as well as in cancer therapy or treatment of bone cancer with physiological bone remodeling [4].

The porous polyhydroxybutyrate/chitosan (PHB/CHIT) polymer is a promising scaffold for bone tissue engineering [5]. Polyhydroxybutyrate (PHB) is a member of the polyhydroxyalkanoates, the biodegradable polyesters, which have been used for different biomedical applications, such as sutures, repair devices and patches, stents, articular cartilage, bone marrow scaffolds, etc. The product of degradation of PHB is the 3-hydroxybutyric acid, a product similar to glycolic acid and lactic acid found as a natural metabolite in brain, heart, lungs, liver, and muscular tissue. An important feature of the PHB is that it offers the necessary mechanical properties, which is the top priority for hard tissue applications [6]. Disadvantages of using this polymer as biomaterial are its hydrophobic nature, low thermal stability, low degradation time, and brittleness. PHB can be physically or chemically blended with other polymers, chitosan (CHIT), and its properties can be improved [7,8,9]. CHIT is a biopolymer derived from chitin, a natural component of the cell wall of fungi and the exoskeleton of arthropods. CHIT consists of a ß-(1–4)-linked d-glucosamine and a *N*-acetyl-d-glucosamine [10]. Its biocompatibility, biodegradability, non-toxicity, antioxidant, antibacterial, anticancer, and anti-inflammatory activities make this biopolymer attractive for tissue engineering [11,12,13,14]. However, it is not likely that CHIT alone can be used to make the scaffold structure. Its use is seriously limited because of its insolubility in water and most organic solvents, faster depolymerization in the body, blood incompatibility, and poor mechanical strength. CHIT also does not meet the mechanical requirements of the implant site. Therefore, it is necessary to combine CHIT with other polymers to improve its physical and chemical properties [15,16,17]. The original porous PHB/CHIT biopolymer scaffold investigated in the present study combines the mechanical features of PHB with the chemical features of CHIT to achieve the required functional results for hard tissue regeneration. PHB/CHIT was successfully tested in vitro for the promotion of chondrogenic activity of mesenchymal stem cells, and preliminary in vivo testing in sheep has shown its applicability for the treatment of chondral and osteochondral defects [8,18]. Eventually, the high level of osteo-conductivity and low level of osteo-inductive activity of CHIT can be supported by osteo-inductive capabilities of PHB [19].

Angiogenesis plays an important role in the development, tissue repair, and wound healing and is also one of the main factors for the safe and successful use of biomaterials [20]. The establishment of a functional vascular network within tissue engineered scaffolds is required to provide a sufficiently perfused environment for new tissue growth and maturation [21]. In addition to delivering nutrients, growth factors, minerals, and oxygen for tissue restoration, this vascular bed provides a transport route for mesenchymal stem cells to facilitate regeneration [22]. Angiogenesis is controlled by the multiple growth factors, endocrine and paracrine molecules, with precise spatial and temporal regulatory activity [23]. A leading role among these factors play a vascular endothelial growth factor (VEGF), fibroblast growth factor-2 (FGF-2), angiopoietins (ANG-1 and ANG-2), and vascular endothelial cadherin (VE-CAD) [23,24,25]. Hence, there is an urgent need for pre-clinical studies to the process of angiogenesis within engineered tissue constructs.

Avian embryos, especially their chorioallantoic membrane (CAM), can be used as a valuable pre-screening assay to determine the angiogenic potential and initial tissue responses of graft materials before more extensive in vitro studies in mammals, especially in the case when multiple experimental conditions need to be tested [26]. The avian CAM is an extraembryonic membrane formed on the fourth embryonic day (ED4) by the fusion of two extraembryonic membranes, the chorion and the allantois [1,5,27,28]. The major functions of the CAM are to support extraembryonic respiratory capillaries, transport sodium and chloride to the allantoic cavity and calcium from the eggshell, and maintain the acid-base balance [29,30]. The CAM consist of three layers: the chorionic epithelium (ectoderm) consisting of villous cavity cells, capillary covering cells, and endothelial (basal) cells, the mesenchyme (mesoderm), and the allantoic epithelium (endoderm), which consists of a single layer of squamous fibroblast-like cells with filopodia. The mesenchymal layer is formed by the fusion of the splanchnic mesoderm of the chorion and the splanchnic mesoderm of the allantois. This double-layer contains large, rapidly growing blood vessels and stroma [27,31,32,33]. The dense vascular plexus of the CAM of chicken (*Gallus gallus domesticus*) embryos, make it a widely used in vivo assay in the fields of basic angiogenesis research, bioengineering, tissue transplantation, oncology, and genomics [27,29,34,35]. The use of this model can reduce the need for mammal models or replace them in scientific research; therefore, it is considered a successful animal model for implementation of the 3R principles [29]. Chicken embryos are also exempted from the European horizontal legislation on the protection of animals used for scientific purposes (EU Directive 2010/63/EU for animal experiments) [1,36].

In recent years, the CAM has specifically become a popular assay in tissue engineering studies [2,24,37]. The avian embryo system is naturally immunodeficient during its early development and thus provides a valuable tool to study the initial tissue response and angiogenic response to biomaterials and xenografts [33,38,39,40]. Using this animal model, angiogenic activity and biocompatibility have been investigated in response to various materials, such as bioglass [41], biphasic hydroxyapatite ceramics [42], medical grade nylon and silastic/silicone tube [43], poly-L-lactic acid [44], or poly(glycerol sebacate urethane) [45], also growth factors, cytokines, hormones, drugs, tissue extracts, and implants. Zwaldo-Klarwasser et al. [34] suggested that materials differ in their ability to influence the angiogenic response of the CAM due to the different chemical composition of the individual materials. Homogeneous materials stimulate angiogenesis (e.g., filter paper), and non-homogeneous materials inhibit angiogenesis (e.g., collagen) [26,34].

In our previous study [5], we provided proof of concept for the use of the CAM model to study PHB/CHIT scaffolds. Herein, we used this model to quantitatively investigate angiogenesis potential of PHB/CHIT scaffold in different conditions, with angiogenic growth factors and in the presence of tyrosine kinase inhibitor using the chicken CAM assay.

## 2. Materials and Methods

### 2.1. Preparation and Characterization of Composite Scaffold

The polyhydroxybutyrate/chitosan scaffold (PHB/CHIT) was prepared according to the method of Medvecky et al. [46], and the analysis was conducted as previously reported [7,46,47] Polyhydroxybutyrate (PHB, GoodFellow, Cambridge, UK) dissolved in propylene carbonate (1% solution of PHB) and chitosan (CHIT, Sigma Aldrich, MO, USA, 1% solution in 1% acetic acid) were mixed together in a 1:1 ratio using a magnetic stirrer (10 min, 400 rpm). After 10 min of mixing, 5 mL of acetone was added to the suspension to achieve precipitation of biopolymers. Final blends were then filtered, washed with distilled water, and compressed into a larger block (4 × 25 × 1 mm), which was then cut into smaller pieces with the final dimensions of 4 × 4 × 1 mm and lyophilized (Freeze dryer, IlShin Biobase Europe, Ede, The Netherlands) for 6 h. The swelling of the composite samples was measured in 1.5 mL vials by immersion of porous substrates (approximately 20 mg) to 0.9% NaCl solution at 37 °C up to a constant mass. Soaking was conducted in triplicate and swelling was evaluated as the ratio of weight of the wet sample to the original dry sample. The microstructure of scaffolds was observed by scanning electron microscopy (FE SEM JEOL7000). The phase analysis of blend was evaluated using the X-ray powder diffraction analysis (XRD, Philips X Pert Pro). The average molecular weights of both used polymers in blends were determined by GPC at level 80 and 28 kDa, respectively. The macroporous and spongy-like microstructures with unsymmetrically shaped macropores with sizes up to 80 µm (approximately 5%) and wide distribution of micropores (less than 30 µm; 90%) were composed of the larger plate-like particles characteristic for CHIT and the fine microporous agglomerates with both the fibrous and more granular morphologies representing PHB. The material used in our study is non-toxic, and no toxic solutions were used in its preparation and production. The tested porous scaffold was sterilized in an autoclave before the application to the CAM surface.

### 2.2. Chick CAM Ex Ovo Model for Evaluation of the Biocompatibility and Angiogenic Response to Biomaterials

Fertilized chicken eggs (*Gallus gallus domesticus*, Lohmann Brown breed, *n* = 194) were purchased from the chicken farm (Párovské Háje, Nitra, Slovak Republic) and delivered in a temperature-controlled manner to ensure egg viability and quality. The eggs were incubated horizontally in a forced-draft constant-humidity incubator at 37.5 ± 0.5 °C and 60% relative humidity. At embryonic day (ED) 3, the eggshell was disinfected with 70% ethanol, each egg was cracked, and the content with chicken embryo was carefully transferred into a plastic weighing boat. The embryos were incubated until ED7 in a still draft incubator (37.5 ± 0.5 °C, 70% relative humidity).

On ED7, the sterilized porous scaffold (PHB/CHIT; 2 × 2 × 1 mm) was carefully placed on the CAM surface alone or soaked with saline solution (PHY, Sodium Chloride 0.9%) and growth factor (VEGF-A, Thermo Fisher Scientific, Waltham, MA, USA; application dose of 25 ng) was prepared by dilution in sterile 0.1% BSA in PBS (Sigma-Aldrich, St. Louis, MO, USA). Similarly, scaffold was soaked with tyrosine kinase inhibitor (SU5402, Sigma-Aldrich, St. Louis, MO, USA) in 5 mM concentration (1.5 mg of SU5402 was dissolved in 1 mL DMSO, Sigma-Aldrich, St. Louis, MO, USA) and placed on the CAM. Implantation site of the tested scaffolds was chosen according to the main conditions of the CAM assay halfway between the embryo and outer border of the CAM and between two large vessels. Three days after the implantation (ED10), the samples of the CAM-PHB/CHIT complex were excised, leaving a margin of 0.2 cm CAM around the scaffold for histological and immunohistochemical examination and borderless CAM for molecular analysis.

### 2.3. Macroscopic Evaluation of Angiogenic Response

The macroscopic evaluation of the angiogenic response was performed using the vascular index. The vascular index was measured as a difference between the number of vessels in the surrounding area of the implants at the beginning of treatment (ED7) and the number of vessels 72 h after implantation (PHB/CHIT: *n* = 25, PHB/CHIT+PHY: *n* = 20, PHB/CHIT+VEGF-A: 24, PHB/CHIT+SU5402: *n* = 24). The CAM blood vessel formation was observed using a stereomicroscope Olympus SZ61 (Olympus, Tokyo, Japan) and digital camera PROMICAM 3-3CP (software QuickPHOTO MICRO 3.2, Prague, Czech Republic). From each biological replicate, a single macroscopical image was taken. The vessel counts were performed using the ImageJ software, Cell Counter Plugin (ImageJ 1.53e, National Institutes of Health, Bethesda, MD, USA). Images were first converted to grayscale (8-bit), sharpened, and then all discernible vessels growing toward the scaffold were counted manually. All experimental procedures were repeated three times.

### 2.4. Histological Examination

Histological examination was performed from the PHB/CHIT scaffold with surrounding CAM tissue. After the fixation in Dent´s solution, the samples were dehydrated in ethanol series and embedded in paraffin. The specimens were serially cut at 7-μm using a rotary microtome (Leica RM2244, Leica Biosystems, Deer Park, IL, USA). The samples were deparaffinized and hydrated with distilled water followed by staining in Mayer’s hemalum solution (Millipore Sigma, St. Louis, MO, USA). Subsequently, sections were rinsed and stained with Eosin (Sigma-Aldrich, St. Louis, MO, USA). Samples were dehydrated in ethanol series and mounted in a permanent medium (Entellan, Millipore Sigma, St. Louis, MO, USA). All stained samples were evaluated by two independent researchers using a light microscope Olympus CX43 (Olympus, Tokyo, Japan) and digital camera PROMICAM 3-5CP+ (Promicra, Prague, Czech Republic) at 20× magnification.

#### Morphometric Analysis

The morphological analysis was performed by two independent researchers using the microscope Olympus CX43 (Olympus, Tokyo, Japan) and digital camera PROMICAM 3-5CP+ (software QuickPHOTO MICRO 3.2; Promicra, Prague, Czech Republic). Morphometric analysis was performed on CAM of 6 random specimens for each group (*n* = 6 implants for each group). We evaluated the number and diameter of the vessels and thickness of CAM layers using serial H-E sections of the CAM-PHB-CHIT complex prepared under the same conditions, based on stereological principles and morphometric analysis. The number of the vessels was counted in 6 fields of view using 20× magnification 3 times for each section. All detected vessels were measured in 5 directions. After that, they were divided into three groups depending on their diameter (up to 50 µm, up to 100 µm, and above the 100 µm). The thickness of the CAM layers was measured 5 times in 6 sections for each layer and group.

### 2.5. Immunohistochemical Analysis

The formation of vessels in the surrounding area of the scaffold as well as in the pores of the implant was evaluated using the markers of embryonic endothelium (WGA, SNA), myofibroblasts (α-SMA), and macrophages (KUL-01) with immunohistochemical staining.

Sections were incubated with lectins (Wheat Germ Agglutinin, WGA, Invitrogen, Ltd., Paisley, UK; Sambucus nigra (Elderberry Bark) lectin, SNA, Thermo Fisher Scientific, Waltham, MA, USA) coupled with the Alexa 488 dye at 1:50 concentration (diluted in 1% BSA in 0.1% PBS-Triton-X, Sigma-Aldrich, St. Louis, MO, USA) for 60 min at room temperature (RT). Nuclei were counterstained with HOECHST (1:80,000, diluted in 0.1% Triton-X in distilled water, Sigma-Aldrich, St. Louis, MO, USA) for 10 min. Slides were dehydrated and mounted in Vectashield medium (Vectashield Antifade Mounting Medium, Vector Laboratories Inc., Newark, CA, USA).

The presence of the myofibroblasts was evaluated using primary monoclonal mouse antibody α-SMA (1:800, Sigma-Aldrich, St. Louis, MO, USA) applied overnight at +4 °C. The sections were then washed in PBS, and TRITC-conjugated goat anti-mouse TRITC (1:200, Jackson ImmunoResearch Laboratory, West Grove, PA, USA) was applied for 90 min in the dark at RT. The sections were dehydrated in ethanol series and mounted in Vectashield medium (Vectashield Antifade Mounting Medium, Vector Laboratories Inc., Newark, CA, USA).

For the evaluation of the macrophages, the PHB/CHIT scaffolds with surrounding CAM tissue were treated with 30% rabbit serum (Sigma-Aldrich, St. Louis, MO, USA). The chicken macrophages were detected by adding mouse anti-chicken monocyte/macrophage primary antibody clone KUL-01 (1:800, Southern Biotech, Birmingham, AL, USA) for 1 h at RT. Slides were counterstained with Mayer’s hemalum solution (Millipore Sigma, St. Louis, MO, USA), dehydrated, and mounted with DPX (Sigma-Aldrich, St. Louis, MO, USA). Images were taken using an upright microscope Olympus CX43 (Olympus, Tokyo, Japan) and digital camera PROMICAM 3-5CP+ (software QuickPHOTO MICRO 3.2; Promicra, Prague, Czech Republic) at 40× magnification to allow visualization of macrophages.

Fluorescently stained sections of the chicken CAM with implanted PHB/CHIT porous scaffold were examined and documented using the fluorescence microscope Olympus BX51 (Olympus, Tokyo, Japan) and digital camera Olympus DP80-U-TV1X-2 T7 (software cellSenseStandard).

### 2.6. Gene Expression Analysis

The biomaterial was collected from CAM using scissors for molecular analysis on the ED10. The analysis was conducted as previously reported [48]. Total RNA was extracted from biomaterial using QIAshredder and total Rneasy Mini Kit from Qiagen (Qiagen, Hilden, Germany) following the manufacturer´s instructions including genomic DNA digestion using the RNase-free Dnase set (Qiagen, Germantown, TN, USA). The RNA purity and yields were analyzed using the NanoDrop Lite Spectrophotometer (Thermo Fisher Scientific, Waltham, MA, USA). We used two-step RT-qPCR approach. In the first step, complementary DNA (cDNA) synthesis was performed using a protocol for RT2 First strand Kit (Qiagen, Germany). A total of 1 µg of total RNA was used to prepare 20 µL of cDNA, which was then used for qPCR. In the second step, the quantification of genes of interest in the cDNA samples was performed using specific primers for VE-Cadherin, Angiopoietin-2, and VEGF-A [49]. For each gene, SYBR Green Mastermix (Qiagen, USA) was used in a total volume of 25 µL. PCR mixture contained specific primers for each gene (300 nM), SYBR Green PCR MasterMix, and water. cDNA for GAPDH was used as an endogenous control for calculating fold differences in RNA levels by the 2-ΔΔCT method. qPCR was performed under the same conditions for SYBR Green with the following steps: Initialization at 95 °C for 10 min, amplification in 40 cycles at 95 °C for 15 s followed by 60 °C for 1 min. Dissociation curve analysis was performed after each completed PCR run to insure the absence of nonspecific amplifications. The gene expression data were calculated against GAPDH endogenous control and expression levels of selected genes were normalized to untreated samples (control).

### 2.7. Statistical Analyses

The statistical analysis was performed by using one-way ANOVA with Sidak´s multiple comparisons test and two-way ANOVA with Dunnet´s multiple comparisons tests using GraphPad Prism 9.3.1 software (GraphPad Software, LLC, San Diego, CA, USA). All measurements were reported as mean ± standard deviation (SD) of *n* = 3 independent experiments. The differences were considered significant at *p* < 0.05.

## 3. Results

### 3.1. Macroscopic Evidence of Angiogenic Response

**Figure 1:**
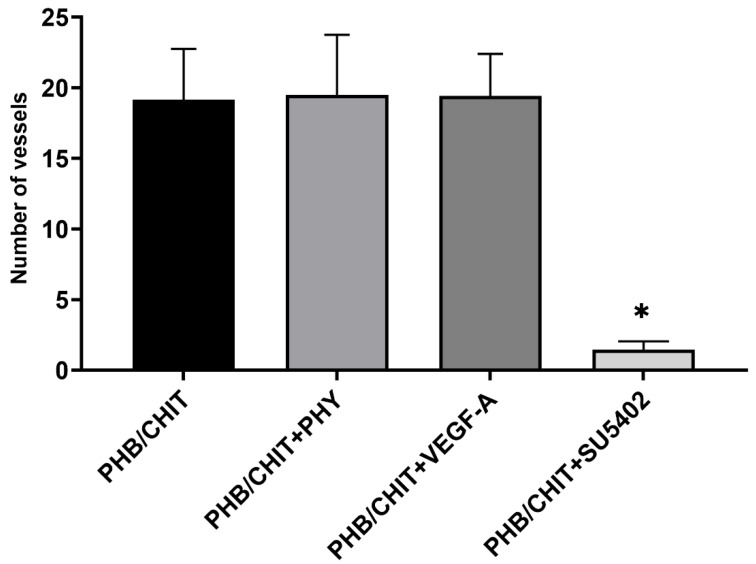
Macroscopic evidence of angiogenic response and in vivo angiogenic activity of PHB/CHIT. Comparison of the average number of newly formed blood vessels in the surrounding area of untreated PHB/CHIT scaffold, scaffold soaked with saline (PHB/CHIT+PHY), vascular endothelial growth factor (PHB/CHIT+VEGF-A), and tyrosine kinase inhibitor (PHB/CHIT+SU5402) showed approximately the same angiogenic activity of scaffolds soaked in saline solution and scaffolds soaked in VEGF-A compared to untreated PHB/CHIT scaffolds. Experiments were repeated three times, and data are shown as mean ± SD (biological replicates: PHB/CHIT: *n* = 25, PHB/CHIT+PHY: *n* = 20, PHB/CHIT+VEGF-A: *n* = 24, PHB/CHIT+SU5402: *n* = 24; technical replicates: 2 images per each biological replicate, one after the implantation and one 72 h after implantation). * *p* < 0.0001.

The macroscopic evaluation of angiogenic response and in vivo angiogenic activity of tested biomaterials showed almost the same angiogenic potential in untreated scaffold compared to soaked scaffolds with pro-angiogenic factor VEGF-A and saline solution (PHY). A significant decrease in the average number of newly formed blood vessels was observed in the scaffold soaked with tyrosine kinase inhibitor (SU5402) compared to untreated PHB/CHIT scaffold and scaffold treated with VEGF-A, PHY as well (Table 1 and Figure 1).

**Figure 2:**
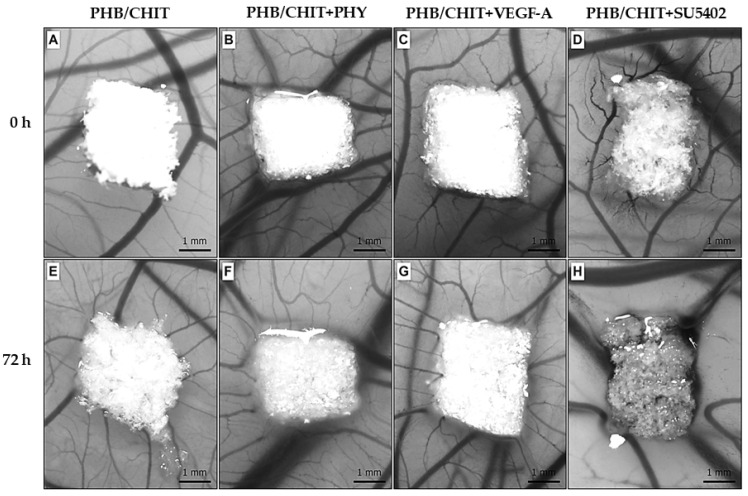
The growing blood CAM vessels toward the scaffolds at the time of implantation (ED7, 0 h) (**A**–**D**) and 72 h (ED10) after implantation (**E**–**H**); scale bar: 1 mm.

The reaction of the CAM immediately after the implantation (0 h) of the porous scaffold and 72 h after the implantation is shown in Figure 2.

The superficial properties of the tested biomaterial allowed it to adhere to the CAM surface and preserve the application site during the whole duration of the experiment.

### 3.2. Histological Evaluation of Angiogenic Response

**Figure 3:**
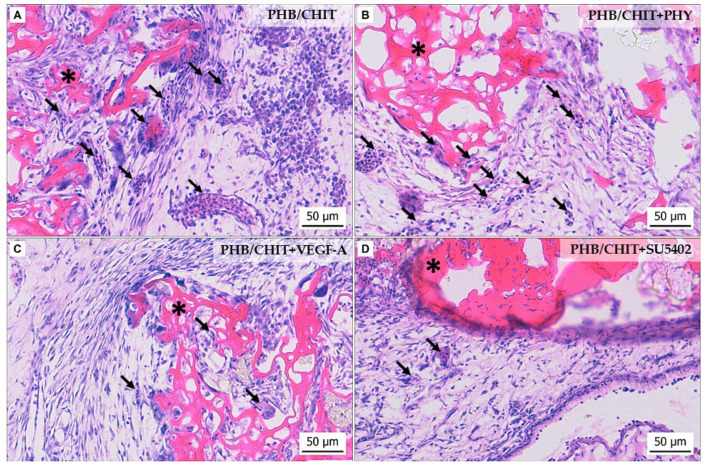
Histological evaluation of the angiogenic response of the CAM tissue 72 h after the implantation of the PHB/CHIT scaffold (**A**) depending on the treatment with PHY (**B**), VEGF-A (**C**) and tyrosine kinase inhibitor (SU5402) (**D**); PHB/CHIT scaffold (asterisk) implanted on the top of the CAM; the vessels of the CAM growth into the material (arrow), staining: H-E; scale bar: 50 µm.

Histological evaluation of the CAM-PHB/CHIT complex showed differences in the morphology of the CAM tissue in the surrounding area of the scaffold depending on the treatment we used (Figure 3). The biomaterial was quite well incorporated with the CAM in all cases. The formation of CAM villi (newly formed CAM tissue) was observed in all samples except PHB/CHIT+SU5402. Hyperplasia of the CAM tissue was observed in all cases.

We observed and evaluated the morphological parameters of the CAM in the surrounding area of the porous scaffold–the number and the diameter of the vessels as well as the thickness of the CAM layers.

#### 3.2.1. Number of Vessels

**Figure 4:**
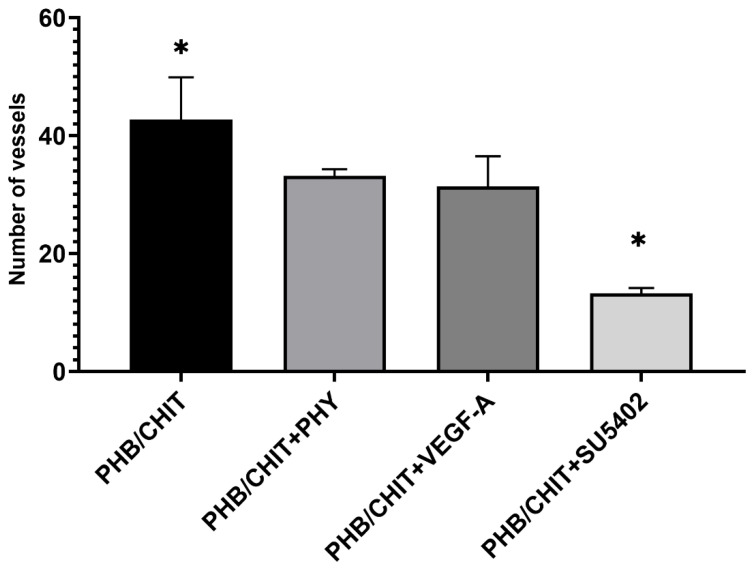
Number of the vessels in the surrounding area of the PHB/CHIT scaffold depending on the treatment of the material. The untreated porous scaffold showed the strong endogenous angiogenic potential while the treatment of the scaffold in the VEGFR-2/FGFR-2/PDGFR-β inhibitor (SU5402) led to a significantly lower number of the vessels around the PHB/CHIT. Experiments were repeated three times, and data are shown as mean ± SD (biological replicates: 6 samples for each group, technical replicates: 120 for each group). * *p* < 0.0001.

According to the number of vessels in the surrounding area of the biomaterial 72 h after the implantation, significantly higher angiogenic potential was observed in the untreated scaffolds (PHB/CHIT, 42.72 ± 7.18) compared to treated scaffolds. Treating the scaffold with saline (33.22 ± 1.11) and the pro-angiogenic factor VEGF-A (31.44 ± 5.07) led to a significantly lower number of vessels compared to PHB/CHIT. The weakest angiogenic potential was observed after the addition of the SU5402 inhibitor (13.28 ± 0.89; Table 2 and Figure 4).

#### 3.2.2. Diameter of Vessels

**Figure 5:**
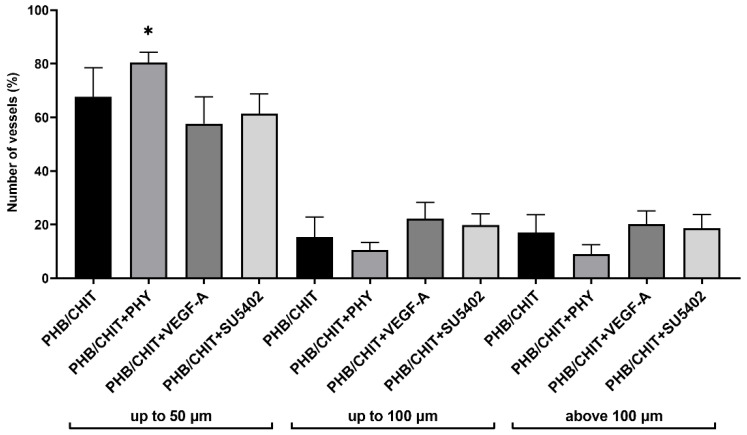
The percentage number of blood vessels of the CAM tissue around the PHB/CHIT scaffolds depending on the diameter of the vessels and treatment of the scaffold. Experiments were repeated three times, and data are shown as mean in % ± SD (biological replicates: 6 samples for each group, technical replicates: 120 for each group). * *p* < 0.0001.

The vessels found in the nearby area of the scaffolds were measured and divided according to their diameter into three groups: vessels with diameter up to 50 µm, vessels with diameter up to 100 µm, and vessels with diameter above the 100 µm. Depending on these conditions, the most represented group of vessels were vessels up to 50 µm (PHB/CHIT+PHY 80.45 ± 3.78%, PHB/CHIT 67.68 ± 10.83%, PHB/CHIT+SU5402 61.45 ± 7.28%, PHB/CHIT+VEGF-A 57.66 ± 9.93%), which may be the sign of ongoing neovascularization. The treatment of the PHB/CHIT with VEGF-A lightly stimulated the growth of the vessels with diameter up to 100 µm compared to the scaffold soaked with PHY (Table 3 and Figure 5).

#### 3.2.3. Thickness of the CAM Layers

**Figure 6:**
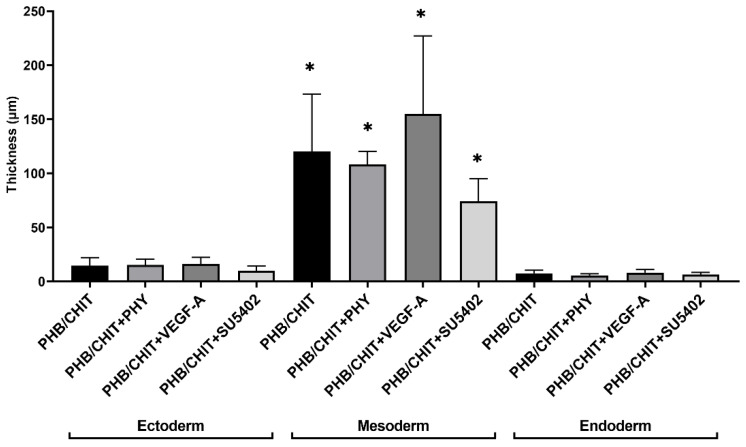
Thickness of the CAM layers in the surrounding area of the scaffold depending on the treatment of the scaffolds. Experiments were repeated three times, and data are shown as thickness of the CAM layers in µm ± SD (biological replicates: 6 samples for each group, technical replicates: 120 for each group). * *p* < 0.0001.

The morphometric analysis of the thickness of the CAM layers showed that the middle mesodermal layer of the CAM was thicker in untreated PHB/CHIT scaffolds as well as in treated scaffolds with PHY, VEGF-A, and SU5402 compared to thickness of ectodermal and endodermal layers of the CAM in all cases. A significantly higher thickness of the mesoderm was observed in scaffold soaked with VEGF-A (154.96 ± 72.11 µm) compared to untreated scaffold (120.22 ± 52.81 µm) and scaffold soaked with PHY (108.06 ± 12.24 µm) and SU5402 (74.33 ± 20.61 µm; Table 4 and Figure 6).

### 3.3. Immunohistochemical Analysis

The immunohistochemical analysis was performed using the samples of CAM-PHB/CHIT complex for detection of specific markers for confirmation of ongoing angiogenesis and ingrowth of newly formed blood vessels inside the pores of the biomaterial, as well as the reaction of the CAM tissue around the biomaterial.

**Figure 7:**
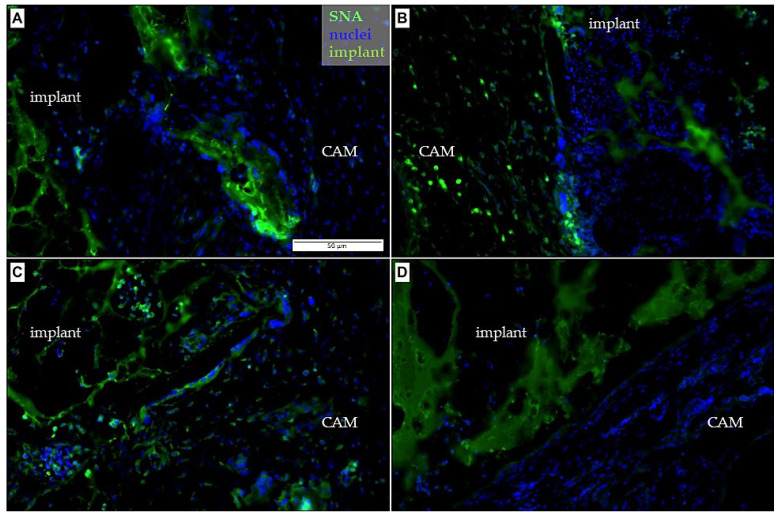
Immunohistochemical detection of the embryonic endothelium in the porous PHB/CHIT scaffolds using the SNA marker. (**A**–**D**) The presence of the SNA positive cells in the surrounding area of the porous scaffolds as well as inside of the pores of the PHB/CHIT was observed suggesting presence of endothelial cells of blood vessels. Treating of the scaffold in tyrosine kinase inhibitor (**D**) led to lower incidence of endothelial cells in the surrounding area and to absence of these cells inside the pores; scale bar: 50 µm for each picture; biological replicates (6 for each group), technical replicates (48 for each group).

In sections of the CAM-PHB/CHIT complex, we observed the SNA positive cells in the surrounding area of the scaffolds as well as inside of the pores of the scaffolds suggesting the presence of endothelial cells in the blood vessels (Figure 7). The detection of endothelial cells and blood vessels by the WGA marker was more complicated due to higher autofluorescence of the scaffold compared to SNA. WGA and SNA stained the vascular endothelium of capillaries, arteries, and veins in the entire vascular bed. Epithelial cells from the CAM ectoderm were observed close to the PHB/CHIT scaffold as well as inside of the scaffold, suggesting good biocompatibility and bioactivity of the biomaterial.

**Figure 8:**
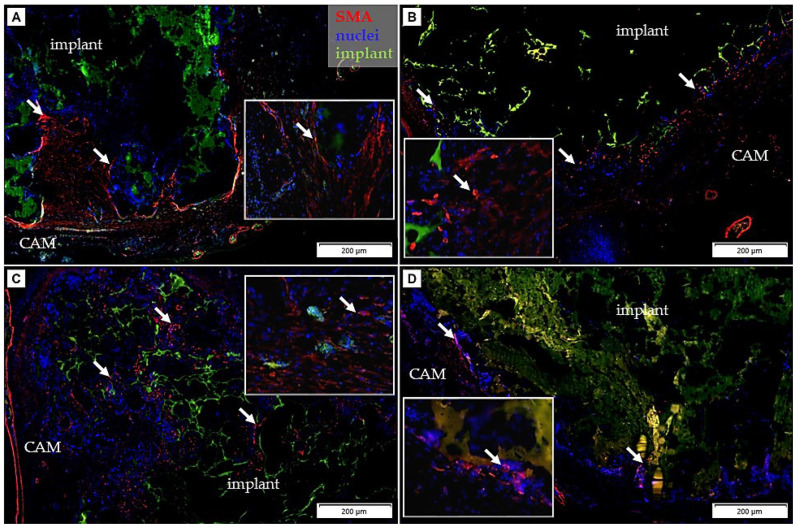
Immunohistochemical detection of the α-smooth muscle actin (α-SMA) in the porous PHB/CHIT scaffolds. The presence of the myofibroblasts (arrow) was observed on a border between the scaffold and surrounding CAM tissue in all cases. The expression level of the α-SMA suggested presence of myofibroblasts in view to ongoing tissue repair; scale bar: 200 µm; biological replicates (6 for each group), technical replicates (48 for each group).

In the surrounding area of the implants, the strong smooth muscle actin reaction using the α-SMA antibody (Figure 8) was detected. Expression levels of the α-SMA suggested the presence of myofibroblasts, which plays a key role during tissue repair and inflammation.

**Figure 9:**
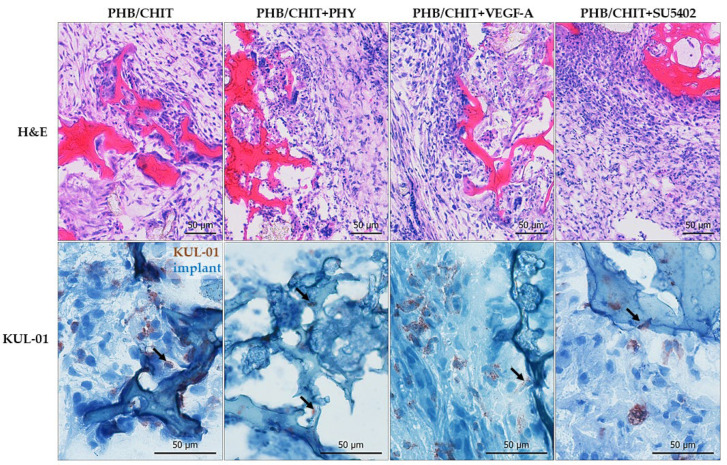
Immunohistochemical detection of the macrophages in the porous PHB/CHIT scaffold using the KUL-01 marker. Immunohistochemistry shows KUL-01 positive cells (arrow) in all tested groups in the surrounding area of the scaffold as well as inside of the scaffold. The presence of macrophages in the pores of the PHB/CHIT scaffolds and surrounding CAM tissue is a sign of ongoing neovascularization (H-E staining; scale bar: 50 µm; biological replicates per group: *n* = 6, technical replicates per group: *n* = 48).

The presence of the macrophages was identified by using the KUL-01 antibody in all of the tested groups (Figure 9). Chicken embryonic macrophages play an important role in angiogenesis. Earlier study with the chicken CAM model indicated the importance of macrophages for neovascularization of implants on the CAM [28]. We conclude that their presence is a sign of new vessels formation in the surrounding area of implanted PHB/CHIT scaffold.

By application of the mentioned markers, we were able to show the evidence of the blood vessels ingrowth to the PHB/CHIT biomaterial, suggesting that angiogenesis takes place in the scaffolds.

### 3.4. Gene Expression Analysis

In general, we could notice upregulation and downregulation of genes of interest based on the used biomaterials. We compared RNA levels of the biomaterial (PHB/CHIT) treated by three different types of solution (PHB/CHIT in a saline solution, PHB/CHIT in a VEGF solution and PHB/CHIT in tyrosine kinase inhibitor SU5402) to RNA levels of control sample (dry PHB/CHIT) (Table 5).

For VEGF-A gene, a statistically significant downregulation was observed in PHB/CHIT submerged to tyrosine kinase inhibitor SU5402 (up to 0.23-fold). In PHB/CHIT submerged in VEGF, the gene expression was slightly downregulated (0.97-fold) and the gene expression of VEGF in PHB/CHIT submerged in a saline solution the gene expression was on the same level as the control sample (1.00).

For Angiopoietin-2, a statistically significant upregulation was observed in PHB/CHIT submerged in VEGF (up to 1.90-fold). Downregulation was present in PHB/CHIT submerged in a saline solution (0.83-fold) and the level of gene expression for PHB/CHIT submerged to inhibitor was the same as in control sample (1.00).

**Figure 10:**
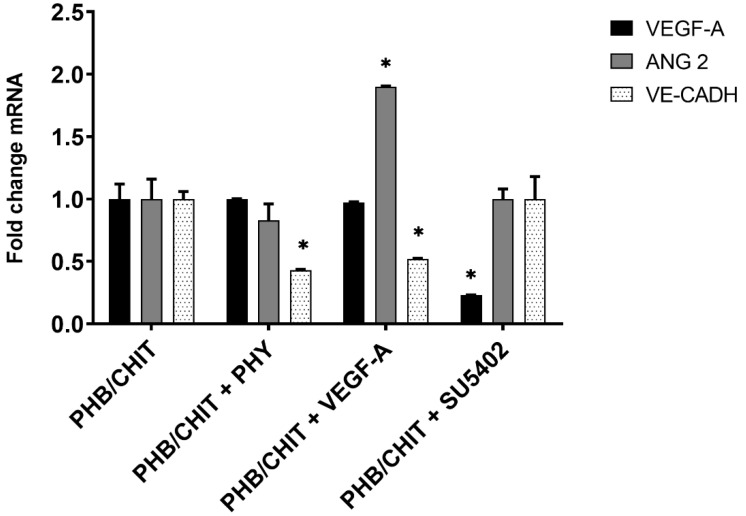
Relative gene expressions from RT-qPCR analysis for genes VEGF-A, ANG-2, and VE-CADH (* statistically significant difference; * *p* < 0.0001). Experiments were repeated three times (biological replicates: 5 samples for each group, technical replicates: 15 for each group). * *p* < 0.0001.

For VE-Cadherin, a statistically significant downregulation was observed. For PHB/CHIT soaked with VEGF-A it was 0.52-fold and for PHB/CHIT soaked with PHY it was −0.43-fold. For PHB/CHIT soaked with inhibitor (PHB/CHIT+SU5402), the level of gene expression was at the same level as in control sample (1.00) (Figure 10).

## 4. Discussion

Different natural or synthetic biomaterials are made to serve as cell or drug carriers (e.g., anticancer drug delivery system) as well as to generate 3D scaffolds, which can be implanted into the human body to replace, remodel, regenerate, or support damaged tissue or organs and to support or improve the self-healing process [45,50]. The avian CAM offers a reproducible and technically simple bioassay for the study of their angiogenic response and biocompatibility, which are crucial for tissue engineering. It is specifically useful to test multiple experimental conditions at a relatively high throughput compared to other animal models [26,30,41].

Biocompatibility of the material is evaluated in terms of the angiogenic response of the CAM in relation to the implanted material, based on a macroscopic evaluation of vascular formation at the implantation site. The proangiogenic effect is manifested by the formation of new blood vessels from pre-existing vessels [38,51]. Biomaterials differ greatly in their ability to initiate an angiogenic response in vivo; while some materials promote the neovascularization, others may suppress the formation of blood vessels, mainly due to different surface activity as well as physicochemical characteristics. The anti-angiogenic effect has been described, for example, in the testing of PVC, which was used to make catheter material. Experimentally produced Tecoflex^®^ and HEMA-Tecoflex^®^ materials have also caused the inhibition of angiogenesis [52].

It follows that the angiogenic response is mainly affected by the chemical properties of the biomaterial surface. Fine, homogeneous materials stimulate angiogenesis while more rough, non-homogeneous materials may inhibit angiogenic process. Further important chemical characteristic of the material that affects the biocompatibility and angiogenic potential of tested material is the porosity [18,52]. The porous scaffold represents a suitable area for distribution, adhesion, and proliferation of the cells and tissue ingrowth; the porosity of the material, pore diameter, pore shape, and structure of the porous scaffold may affect the cell expansion and make a difference in angiogenic response and inflammation [18,44,53]. Magnaudeix et al. [54] concluded that the shape of the pores effects the blood vessel guidance, diameter, and number of the vessels while the size of the pores has a dramatic effect on the mobility of the cells and differentiation, which may lead to a change in ingrowth of cells and blood vessels [55]. Low porosity and small pore diameter (less than 26 µm) have a negative effect on cell penetration while reducing collagen production. However, the high porosity of the material (96%) and the medium pore size (26–28 µm) promote tissue and blood vessel ingrowth without the need for additional biochemical stimuli [45]. In our case, the investigated acellular PHB/CHIT shows 85% porosity; 90% of the pores do not reach more than 30 µm in size, and approximately 5% are macropores with a size over 80 μm [56]. In our previous study, we proved the presence of blood vessels inside the innovative acellular porous PHB/CHIT scaffold using the quail (*Coturnix coturnix japonica*) CAM assay. This descriptive methodology-oriented study aimed to verify the utility of the CAM model for testing biocompatibility of innovative porous scaffolds. The presence of a vascular network was confirmed by identifying hemangioblasts and endothelial cells using the QH1 marker [5]. Based on our previous findings, we here tested the angiogenic potential of PHB/CHIT scaffold treated with pro- and anti-angiogenic substances using the chicken CAM model. Our result show that the original porous PHB/CHIT scaffold supports a strong angiogenic response, which is not increased by adding the potent pro-angiogenic factor, VEGF-A, but which is inhibited by using the tyrosine kinase inhibitor SU5402. This data indicated that PHB/CHIT could find use in the field of regenerative medicine as a promising scaffold for treatment of hard tissue defects, especially when a strong angiogenic response is required.

Various qualitative and quantitative methods have been used for determining angiogenesis after the application of different factors or substances on the CAM surface. For quantification of the newly formed vessels in CAM tissue, the vascular index is used to assess the number of new vessels radially growing into the implant [57]. Very useful is to evaluate vascular morphology (macroscopic and microscopic evaluation) performing a morphometric analysis of the vessels based on stereology. As CAM provides a natural environment for neovascularization; the simplest evaluation is to observe the presence or absence of blood vessels and changes in vascular network of the CAM [1,29,35]. The main rapidly growing vessels can be found in mesoderm while the chorionic epithelium consists of a single layer of small vessels [31].

Visualization of the vascular network in the chicken CAM has been met with some difficulties due to the lack of specific reagents to stain endothelial cells. The endothelium of the blood vessels represents a highly specialized semipermeable barrier that contains carbohydrate residues, which can be evaluated using lectins, nonimmune origin proteins specific for carbohydrate commonly used for visualization of blood vessels in several organs and animal species [58,59]. In our case, WGA and SNA were used for visualization of the embryonic blood vessels, labelling the extracellular matrix. WGA bounds well to the luminal surface of vessels; therefore, it can be effectively used as a marker for the visualization of the vessels of early stages of the embryo development [58]. An important role in the body immune response and in developmental angiogenesis of the embryo is undertaken by macrophages, which have been demonstrated to express pro-angiogenic markers [28]. Macrophages are found when angiogenesis and inflammation occur [60]. Macrophages were found in the surrounding area of the implant as well as in the pores of the PHB/CHIT scaffold. The CAM tissue reaction after the implantation is associated with the inflammation and the wound healing process consisting of neovascularization, regeneration, and reparation with fibrosis [35,38,43]. The PHB/CHIT scaffold was surrounded with a fibrotic layer consisting of myofibroblasts, expressing α-SMA, suggesting an ongoing process of wound healing [61,62].

Ongoing tissue regeneration, and angiogenesis as well, is accompanied by changes, which are under control by positive regulators of angiogenesis [25,63].

One of the most important mechanisms involved in the vascular plexus development is regulated by expression of angiogenesis-regulating genes, such as VEGF-A. The angiogenic effect of VEGF-A is initiated through its binding with tyrosine kinases receptors found in vascular endothelial cells (VEGFR-1 and VEGFR-2). The main role of this receptor is to modulate the availability of VEGF to VEGFR-2 as the VEGFR-1 has higher affinity to VEGF [64]. VEGF and VEGFR-2 signaling pathway controls the function of endothelial cells during angiogenesis and in cooperation with FGF-2 stimulates angiogenesis through proliferation of endothelial cells [65]. FGF-1 and FGF-2 represent the most extensively investigated proteins from FGF family, which are implicated in angiogenesis by stimulating the endothelial cells’ mitosis and migration [66]. Studies on the normally developing CAM found an expression peak of VEGF-A at ED13 and ED20 while vascular endothelial growth factor receptor-2 (VEGFR-2) expression was documented at ED11 [32]. Maximal concentration of FGF-2 was observed at ED10 and ED14 [38]. We demonstrated that untreated acellular porous PHB/CHIT has an angiogenic potential, which may have a direct and/or an indirect effect on vascular receptors through activation of VEGF or synergistic activity with VEGF or FGF. The changes in these parameters may be made by sprouting and intussusception [67]. Angiogenic stimulators, such as VEGF and FGF-2, are recommended as a positive control group [68]. In our case, the addition of 25 ng VEGF-A lead to upregulation of ANG-2 gene expression and downregulation of VE-Cadherin gene expression. This indicates a more active state of the blood vessel; however, the expression profile was not linked to a higher vascularization as such.

Angiopoietins are important growth factors for vascular development and quiescence. This ANG family consists of two receptors (TIE-1 and TIE-2) and three ligands (ANG-1, ANG-2, and ANG-4). For the vascular endothelium, only ANG-1 and ANG-2 are specific. No ligand of TIE-1 has been identified; therefore, TIE-2 is considered as negative regulator while TIE-2 plays a role in hematopoiesis and angiogenesis. ANG-2 blocks TIE-2 receptor and acts to repel pericytes and α-SMA positive cells. The function of ANG/TIE-2 pathway seems to be restricted to later stages of blood vessel development, and it is also involved in remodeling and maturation of the vascular network [69,70]. No difference was observed in ANG-2 expression between the different PHB/CHIT scaffolds.

Interestingly, the addition of SU5402 led to a significant downregulation of VEGF-A expression and also to lower angiogenesis. SU5402 used in our study is a potent and selective inhibitor of vascular endothelial growth factor receptor 2 (VEGFR-2) and fibroblast growth factor receptor (FGFR), as well as platelet-derived growth factor receptor beta (PDGFR-β). Future experiments with more selective tyrosine kinase inhibitor should be performed to investigate whether the anti-angiogenic response is mainly driven by VEGF-A inhibition or by inhibition of FGF-2, or PDGFR-β.

In our study, we used standard *ex ovo* techniques to assess the biocompatibility of an acellular porous PHB/CHIT scaffold and PHB/CHIT scaffolds treated with pro- and anti-angiogenic substances as well. This technique is more often used but the viability of embryos is limited. During the first three days, 50% of deaths must be expected [71]. Despite the appropriate conditions for embryo handling, we also recorded 40% to 50% of embryo deaths in the first three days of incubation. However, this technique provides better conditions for interventions. It follows that the vascularization process and biocompatibility of biomaterial can be observed at all times during experiment.

## 5. Conclusions

Our study brings macroscopic, microscopic, and molecular methodological procedures, which allow the observation of angiogenic activity on the chicken CAM model in surrounding area of the implanted scaffold as well as the presence of endothelial cells inside its pores. It assumes good biocompatibility and bioactivity of tested biomaterial. This effect was observed using the acellular PHB/CHIT with 85% porosity. In newly formed CAM tissue as well as inside the scaffold pores, the formation of the CAM villi and the presence of endothelial cells in blood vessels were observed. The presence of myofibroblasts on a border between CAM tissue and the biomaterial as well as the presence of macrophages inside the pores of scaffold is a sign of repair process of the CAM tissue and ongoing angiogenesis.

Investigated acellular PHB/CHIT biomaterial showed a strong endogenous angiogenic potential, which does not need to be increased by adding the potent proangiogenic factor, VEGF-A, and could be a promising material for treatment of hard tissue defects and diseases in the field of regenerative medicine. Another use of PHB/CHIT material can be aimed as a filler of bone defects in tumor disease. It seems to be ideal for further experimental purposes in combination with trophic factors (growth factors, cytokines, chemokines, etc.) and mesenchymal stem cells.

The chicken CAM assay has the potential to become an integral part of pre-clinical testing of biocompatibility and functionality as well as the tissue reaction of potential biomaterials that can be used in regenerative medicine or cancer therapy of hard tissue.

## 6. Patents

There is a patent resulting from the work reported in this manuscript: Lubomir Medvecky, Maria Giretova, Eva Petrovova: Biopolymer composite system for cartilage regeneration: Published patent application no. 89-2014. Banska Bystrica: Industrial Property Office of the Slovak Republic, 9 September 2019.

## Figures and Tables

**Table 1:** The vascular index expressed as difference between number of vessels immediately after the implantation (ED7) and 72 h after the implantation of PHB/CHIT (ED10). Values are means ± SD of *n* = 3 independent experiments.

Treated Material	Total Samples	Vascular Index
PHB/CHIT	25	19.16 ± 3.59
PHB/CHIT+PHY	20	19.50 ± 4.25
PHB/CHIT+VEGF-A	24	19.42 ± 2.98
PHB/CHIT+SU5402	24	1.46 ± 0.59

Key: PHB/CHIT: untreated polyhydroxybutyrate/chitosan; PHB/CHIT+PHY: polyhydroxybutyrate/chitosan soaked in saline solution; PHB/CHI+VEGF-A: polyhydroxybutyrate/chitosan soaked in vascular endothelial growth factor; PHB/CHIT+SU5402: polyhydroxybutyrate/chitosan soaked in tyrosinase kinase inhibitor SU5402.

**Table 2:** Number of the vessels in the surrounding area of the PHB/CHIT scaffolds 72 h after the implantation (ED10). Values are means ± SD of *n* = 3 independent experiments. Experimental groups included *n* = 6 samples of the scaffold for each group.

Treated Material	Number of Vessels
PHB/CHIT	42.72 ± 7.18
PHB/CHIT+PHY	33.22 ± 1.11
PHB/CHIT+VEGF-A	31.44 ± 5.07
PHB/CHIT+SU5402	13.28 ± 0.89

**Table 3:** Diameter of the vessels of the CAM tissue around the PHB/CHIT depending on the treatment of the scaffold. Data are presented as a mean in % ± SD of *n* = 3 independent experiments. Experimental groups included *n* = 6 implants for each group.

Treated Material	Diameter of the Vessels up to 50 µm	Diameter of the Vessels up to 100 µm	Diameter of the Vessels above 100 µm
PHB/CHIT	67.68 ± 10.83	15.34 ± 7.49	16.98 ± 6.67
PHB/CHIT+PHY	80.45 ± 3.78	10.48 ± 2.87	9.07 ± 3.39
PHB/CHIT+VEGF-A	57.66 ± 9.93	22.21 ± 6.02	20.13 ± 4.97
PHB/CHIT+SU5402	61.45 ± 7.28	19.83 ± 4.12	18.73 ± 4.99

**Table 4:** Thickness of the CAM layers. Data are presented as a thickness of each layer in µm ± SD of *n* = 3 independent experiments. Experimental groups included six samples of the scaffolds for each group.

Treated Material	Ectoderm	Mesoderm	Endoderm
PHB/CHIT	14.62 ± 7.49	120.22 ± 52.81	7.51 ± 2.86
PHB/CHIT+PHY	15.26 ± 5.28	108.06 ± 12.24	5.61 ± 1.50
PHB/CHIT+VEGF-A	16.27 ± 6.00	154.96 ± 72.11	8.20 ± 2.77
PHB/CHIT+SU5402	9.91 ± 4.33	74.33 ± 20.61	6.41 ± 1.99

**Table 5:** Relative gene expression from RT-qPCR analysis for genes VEGF-A, ANG-2, and VE-CADH. Experimental groups included five samples of the scaffolds for each group.

	Genes (Fold Change)		
Treated Biomaterial	VEGF-A	ANG-2	VE-CADH
PHB/CHIT (control)	1.00	1.00	1.00
PHB/CHIT+PHY	1.00	0.83	0.43 *
PHB/CHIT+VEGF-A	0.97	1.90 *	0.52 *
PHB/CHIT+SU5402	0.23 *	1.00	1.00

* Statistically significant difference; * *p* < 0.0001.

## Data Availability

The data presented in this study are available on request from the corresponding author. The data are not publicly available due to the fact that these data are published for the first time and authors have no problems to provide them on request.
